# AI-Driven Digital Twin Architecture for Multimodal Prediction and Adaptive Intervention in Cognitive Aging

**DOI:** 10.2196/87768

**Published:** 2026-07-08

**Authors:** Yu Liu, Keming Yan, Siying Ma, Hao Deng, Jian Kong

**Affiliations:** 1 Department of Psychiatry Harvard Medical School Massachusetts General Hospital Boston, MA United States; 2 Microsoft Customer and Partner Solutions (MCAPS) Microsoft Cambridge, MA United States; 3 Department of Anesthesia, Critical Care and Pain Medicine Harvard Medical School Massachusetts General Hospital Boston, MA United States

**Keywords:** digital twin, cognitive aging, multimodal prediction, machine learning, adaptive intervention

## Abstract

Age-related cognitive dysfunction, including mild cognitive impairment and dementia, underscores the need for scalable and personalized predictive models. We present a conceptual artificial intelligence-driven digital twin framework to support early detection, real-time monitoring, and adaptive intervention. The system is structured around 4 core processes: perception, analytics, decision-making, and adaptive feedback, and is organized across 5 functional layers: data acquisition, integration, modeling, reasoning, and application. Multimodal behavioral, physiological, and clinical data are harmonized using Fast Healthcare Interoperability Resources and Observational Medical Outcomes Partnership standards. Predictive modeling uses convolutional and recurrent neural networks, gradient boosting, and reinforcement learning. The framework is designed for cloud-based deployment on platforms that support HIPAA-aligned implementation, including Amazon Web Services and Microsoft Azure, with 7 application modules spanning signal-based and pose-based assessment, personalized mind-body training, cognitive rehabilitation, and disease trajectory simulation. This architecture offers a foundation for precision cognitive care in aging populations.

## Introduction

Rapid demographic aging is reshaping global health landscapes, with the number of individuals aged 60 years and older projected to increase from 901 million in 2015 to 2.1 billion by 2050 [1]. As life expectancy rises, the burden of age-related cognitive dysfunction, including mild cognitive impairment (MCI) and dementia, is increasing sharply, undermining individual quality of life and placing mounting pressures on health care infrastructures worldwide [2]. A systematic review of global studies reports that the prevalence of cognitive impairment among older adults ranges from 5.1% to 41%, with a median of 19% [1].

Despite growing demands, current health care models remain poorly equipped to manage the dynamic, heterogeneous, and progressive nature of cognitive dysfunction. Conventional care approaches, often episodic and clinic-centered, lack the capacity for continuous longitudinal monitoring, personalized risk stratification, or timely intervention [3]. This limitation frequently results in delayed detection of cognitive deterioration, missing the optimal window for early-stage intervention where preventive strategies may be most effective. Addressing these gaps requires innovative frameworks capable of enabling continuous surveillance, individualized cognitive modeling, and adaptive, real-time care strategies across diverse aging populations [4].

Recent advances in artificial intelligence (AI), machine learning (ML), and digital twin (DT) technologies offer promising avenues to meet these emerging needs [5]. AI and ML algorithms have demonstrated considerable capacity to extract latent patterns from complex, high-dimensional health data, supporting early detection of cognitive decline and the development of personalized intervention pathways [6]. Simultaneously, the adaptation of DT frameworks, originally conceived for engineering and industrial systems, into health care settings enables the creation of dynamic, real-time virtual representations of individual patients [7]. These models can continuously simulate health trajectories, forecast risks, and optimize care strategies based on evolving multimodal inputs [8].

Here, we present an AI-driven multimodal DT framework designed to enable continuous, dynamic, and personalized management of cognitive health in aging populations. By integrating real-time physiological, behavioral, and environmental data with adaptive ML-driven modeling and secure, scalable cloud infrastructures, the framework aspires to facilitate early identification of cognitive risk, dynamic intervention refinement, and proactive resilience building. Moving beyond reactive and episodic care models, this conceptual architecture represents a critical step toward establishing precision, sustainable, and equitable cognitive health care for an increasingly aging global society.

### Conceptual Framework

#### Overview

**Figure 1:**
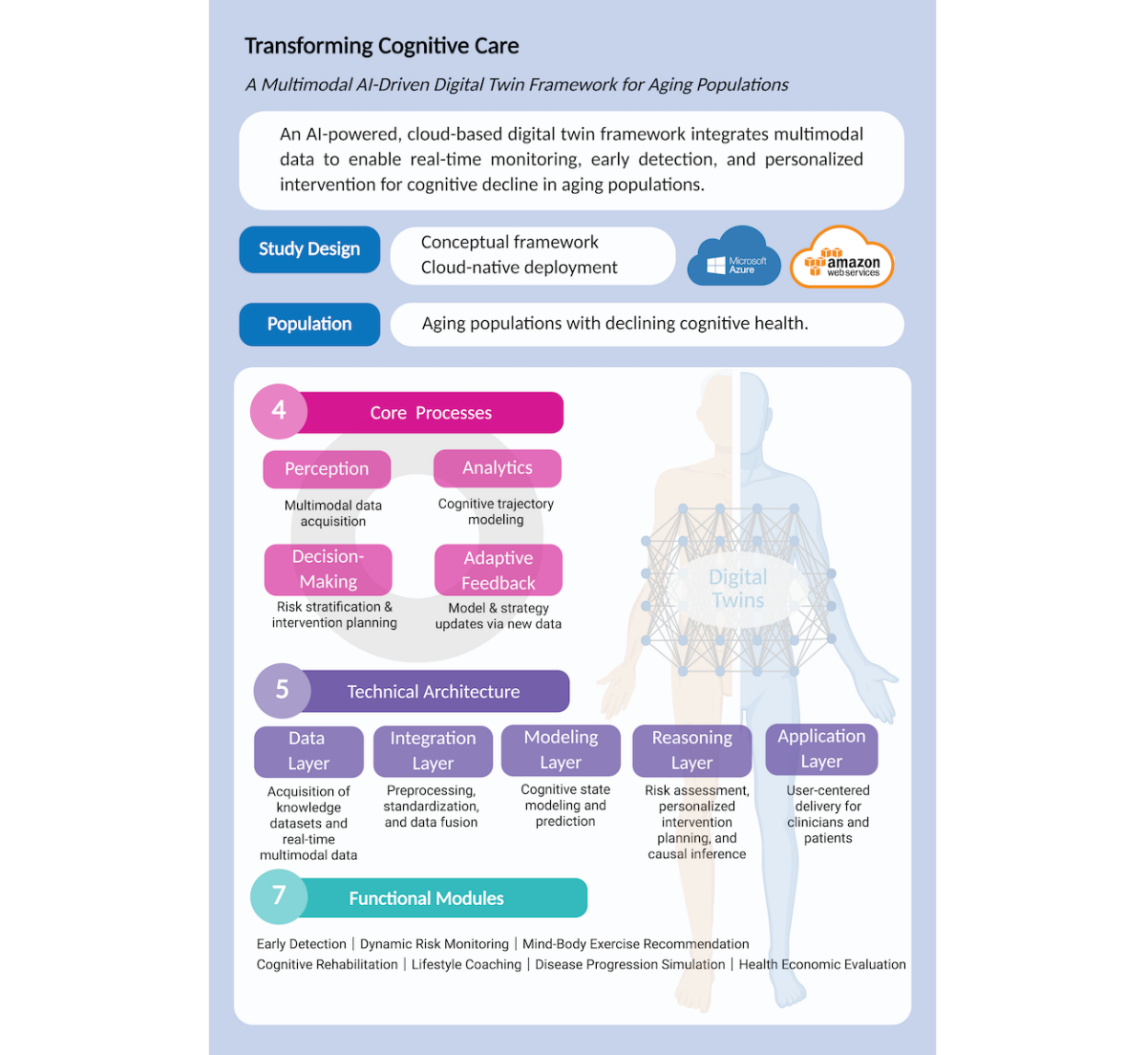
AI-enabled multimodal digital twin framework for personalized cognitive health management in aging populations. AI: artificial intelligence. Created in BioRender [16], licensed under CC BY 4.0 [17].

DT technology, originally developed for engineering and industrial applications, has increasingly attracted attention in health care for its potential to enable dynamic [9], personalized [10], and predictive management of patient health [8]. In the context of cognitive health, a DT is conceptualized as a continuously evolving, real-time virtual representation of an individual’s physiological, behavioral, and environmental states, constructed through multimodal data acquisition, integration, and computational modeling [11]. The AI-driven DT framework proposed in this study is structured around four interdependent core processes: perception [12], analytics [13], decision-making [14], and adaptive feedback [15]. Each process plays a critical role in creating an individualized, dynamic, and responsive model of cognitive function and its evolution over time (Table 1 and Figure 1 [16]).

Figure 1 presents the proposed multimodal digital twin framework, which integrates real-time physiological, behavioral, and environmental data with large-scale clinical and research datasets to enable early detection, continuous monitoring, and adaptive intervention for cognitive decline in older adults. The system is structured around four core processes: perception, analytics, decision-making, and adaptive feedback, operationalized through five technical layers: data acquisition, integration, modeling, reasoning, and application. This structure is consistent with the canonical DT architecture, in which perception aligns with the physical entity, analytics and decision-making are instantiated in the virtual twin, and adaptive feedback is operationalized through the bidirectional communication channel to sustain a closed-loop workflow. Seven functional modules support dynamic, individualized care, including mind-body exercise recommendations, cognitive rehabilitation, lifestyle coaching, disease trajectory simulation, and health economic evaluation. The framework is designed for scalable deployment using cloud-native, vendor-agnostic architectures.

**Table 1:** Core processes of the artificial intelligence–enabled digital twin framework for cognitive health management^a^.

Architecture	Core	Function	Representative components and methods
Physical entity	Perception	Captures and integrates multimodal physiological, behavioral, and environmental data	EEG^b^, HRV^c^, gait, speech, sleep, facial expressions, and environmental sensors; EHRs^d^, ADNI^e^, and UKB^f^; data cleaning, temporal alignment, and FHIR^g^ and OMOP^h^-based harmonization
Virtual twin	Analytics	Extracts latent features and predicts cognitive risk trajectories	CNNs^i^, LSTMs^j^, and autoencoders; multimodal fusion; few-shot learning; uncertainty estimation with Monte Carlo dropout
Virtual twin	Decision-making	Generates individualized intervention strategies based on predictive and causal inference models	Reinforcement learning, Bayesian inference, and propensity score matching; anomaly monitoring; intervention simulation; SHAP^k^ and LIME^l^; personalized mind-body, cognitive, and behavioral recommendations
Bidirectional communication channel	Adaptive feedback	Dynamically updates models and interventions based on new data and user responses	Federated and incremental learning; real-time tracking; edge computing; adaptive tuning of intervention intensity and content; mobile and wearable interfaces

^a^The framework comprises four synergistic processes that transform real-time data into personalized, adaptive interventions for cognitive decline in aging populations.

^b^EEG: electroencephalography.

^c^HRV: heart rate variability.

^d^EHR: electronic health record.

^e^ADNI: Alzheimer’s Disease Neuroimaging Initiative.

^f^UKB: UK Biobank.

^g^FHIR: Fast Healthcare Interoperability Resources.

^h^OMOP: Observational Medical Outcomes Partnership.

^i^CNN: convolutional neural network.

^j^LSTM: long short-term memory.

^k^SHAP: SHapley Additive exPlanations.

^l^LIME: Local Interpretable Model-Agnostic Explanations.

#### Perception

Perception involves the continuous acquisition and preprocessing of diverse multimodal data streams to construct a comprehensive and high-resolution representation of an individual’s cognitive health status [18,19]. Data sources include physiological signals (eg, electroencephalography [EEG] [20], heart rate variability [HRV], heart rate, brain imaging (functional magnetic resonance imaging or magnetic resonance imaging, and positron emission tomography) [21]; cerebrospinal fluid and blood markers, genetic testing [22], behavioral indicators (eg, gait patterns, speech dynamics, and sleep architecture) [23]; facial expression imaging; and environmental exposures (eg, ambient light and noise) [24], collected via wearable devices, mobile apps, and electronic health records. In parallel, knowledge datasets, such as the Alzheimer’s Disease Neuroimaging Initiative [23], UK Biobank [25], peer-reviewed publications, meta-analyses, clinical guidelines, and expert consensus statements, are incorporated to inform normative baselines and support population-level generalizability. All data undergo standardized preprocessing pipelines to ensure quality, semantic harmonization, and interoperability across downstream analytical processes.

#### Analytics

Analytics focuses on the extraction of relevant features, detection of subtle cognitive anomalies, and prediction of future cognitive risks based on synthesized multimodal inputs [26]. Advanced ML models are applied, including lightweight convolutional neural networks (CNNs) for image and speech feature extraction [27], long short-term memory (LSTM) networks for modeling sequential physiological data [28], and autoencoders for unsupervised anomaly detection [29]. The analytics engine constructs individualized cognitive trajectories, enabling early identification of deviations from normative baselines and informing timely, proactive intervention planning.

#### Decision-Making

Decision-making transforms predictive insights into dynamic and personalized intervention strategies [30]. Leveraging a combination of short-term and long-term reinforcement learning (RL) algorithms [30], Bayesian probability estimation frameworks [31], and causal inference techniques such as propensity score matching [31], the system stratifies cognitive risks, simulates potential intervention outcomes, and generates individualized recommendations. These recommendations may include targeted mind-body exercises, cognitive rehabilitation programs, or behavioral and lifestyle modifications tailored to each individual’s evolving cognitive needs [32-36].

#### Adaptive Feedback

Adaptive feedback closes the learning loop by continuously assimilating new data, recalibrating cognitive state models, and refining intervention strategies based on individual responses over time [37]. Federated learning and incremental learning techniques are incorporated to support dynamic model updates while preserving data privacy and minimizing the need for centralized raw data aggregation [38]. This continuous adaptation mechanism ensures that cognitive care remains responsive, personalized, and aligned with real-world changes in patient health status [39].

To align our functional workflow with the canonical DT architecture, we explicitly map the DT’s three structural components: (1) the physical entity, (2) the virtual twin, and (3) the bidirectional communication channel, to the four core processes proposed in this study. Specifically, perception primarily occurs at the physical entity level, where multimodal data are acquired from older adults and their context and undergo initial quality control. Analytics and decision-making are implemented within the virtual twin, which maintains an up-to-date computational representation of an individual’s cognitive and health state for inference, prediction, and intervention selection. Adaptive feedback is enabled by the bidirectional channel that securely transmits state updates and model outputs from the virtual twin back to real-world actions.

## Technical Architecture of the Personalized Cognitive DT Framework

### Overview

To operationalize the AI-driven DT framework for cognitive dysfunction management, we developed a 5-layer technical architecture, encompassing the data layer [40], integration layer [41], modeling layer [42], reasoning layer [43], and application layer [44]. Each layer was designed to perform distinct yet synergistic functions, supporting continuous multimodal monitoring, predictive modeling, individualized intervention optimization, and dynamic adaptation based on real-time feedback (Figure 1).

### Data Layer: Integration of Multimodal Data for Cognitive Health Modeling

The Data Layer enables acquisition, aggregation, and secure management of real-time multimodal data and knowledge datasets critical for constructing individualized cognitive health representations [11]. Real-time data streams are continuously captured across physiological, behavioral, and environmental domains [45]. Physiological signals include EEG, HRV, brain imaging (magnetic resonance imaging, functional magnetic resonance imaging, and positron emission tomography), and heart rate, while behavioral data encompass gait trajectories, speech characteristics, sleep patterns, and facial expression imaging [18]. These data are collected through wearable sensors, IoT devices, and mobile health apps [46].

Brain imaging data, such as hippocampal atrophy, white matter hyperintensities, and resting-state connectivity, provide both structural and functional insights. Cerebrospinal fluid and blood-based biomarkers, including amyloid-β42 (Aβ42), total tau, phosphorylated tau (p-tau181/217), and neurofilament light chain, offer mechanistic specificity. Genetic risk factors, such as the apolipoprotein E epsilon 4 (APOE ε4) genotype, are incorporated to stratify individual risk and inform longitudinal modeling [22]. EEG signals are processed to extract not only conventional event-related potentials but also higher-order complexity metrics, including permutation entropy and spectral entropy, which have demonstrated predictive value for cognitive impairment [47]. HRV features are computed to assess autonomic nervous system function, known to correlate with executive performance and cognitive resilience [48]. Gait variability, a well-established early biomarker of cognitive decline, is monitored using wearable inertial sensors [49]. Speech parameters, including articulation rates, pauses, and prosody, are extracted to detect subtle neurodegenerative changes [50]. Sleep architecture, including sleep efficiency and fragmentation, is captured to evaluate memory consolidation and overall cognitive status [51].

In parallel, knowledge datasets complement real-time acquisitions and include structured outpatient and inpatient electronic health records, large-scale clinical databases such as the Alzheimer’s Disease Neuroimaging Initiative and UK Biobank, as well as peer-reviewed publications, meta-analyses, clinical guidelines, and expert consensus statements [50]. All sources are harmonized through standardized frameworks, including the Fast Healthcare Interoperability Resources (FHIR) and the Observational Medical Outcomes Partnership (OMOP) data models [52]. FHIR primarily supports operational interoperability and clinical data exchange, whereas OMOP is commonly used for harmonized observational analytics. A practical workflow is to ingest clinical data via FHIR, transform to OMOP for modeling, and expose DT outputs back to clinical systems through FHIR resources. To ensure analytical readiness, rigorous data quality assurance procedures, including data cleaning, temporal synchronization, and missing value imputation, are applied across modalities [53]. Privacy and security are safeguarded using differential privacy in aggregation workflows, edge computing for local preprocessing of sensitive data, and end-to-end encrypted transmission protocols to protect information exchange across networks [54] (Figure 1). Required sample size depends on modality coverage, label frequency, and model complexity; the framework supports starting with simpler models in smaller cohorts and scaling to higher-capacity models as longitudinal data accumulate.

### Integration Layer: Standardization and Fusion of Heterogeneous Cognitive Health Data

The Integration Layer enables the standardization, synchronization, and fusion of heterogeneous multimodal data streams into coherent and interoperable feature representations [55]. Temporal synchronization ensures that physiological, behavioral, and environmental data streams are chronologically aligned, preserving the temporal context critical for dynamic modeling [56]. High-dimensional raw signals undergo feature standardization and extraction to transform them into semantically meaningful embeddings [57]. Data fusion is performed at multiple levels: early fusion strategies integrate features at the input stage; late fusion aggregates predictions from independent models; and hybrid fusion merges intermediate latent features for enhanced robustness [58]. Cross-modal autoencoders are used to compress multimodal inputs while preserving critical cross-domain relationships [59]. Feature concatenation techniques further enable unified feature spaces that retain the unique contributions of each modality [60]. Through these processes, heterogeneous data sources are effectively unified to facilitate downstream predictive analytics (Figure 1). Wearable-derived activity features and computerized digital cognitive biomarkers have each been systematically reviewed as feasible, clinically informative signals in older adults, motivating DT fusion pipelines that align heterogeneous modalities on event time and accommodate irregular observation intervals.

### Modeling Layer: Multimodal Predictive Modeling for Personalized Cognitive Trajectories

The modeling layer enables the construction of individualized cognitive profiles and predictive models by synthesizing multimodal information into dynamic risk trajectories [61]. CNNs are deployed to extract spatially localized features from image-like data structures, including EEG topographies, facial expression maps, gait spectrograms, and sleep hypnograms [62]. LSTM networks are used to capture temporal dependencies in physiological time series such as HRV variations and sleep cycle dynamics [63]. Structured tabular data, comprising clinical laboratory results and self-reported cognitive assessments, are analyzed using extreme gradient-boosted trees and support vector machines (SVMs), with SVMs offering advantages for high-dimensional, small-sample settings such as daily cognitive diaries [64]. Multimodal feature fusion is accomplished through cross-modal autoencoders and feature concatenation pipelines, supporting comprehensive cognitive state modeling. Few-shot learning approaches, including Siamese and prototypical networks, facilitate rapid adaptation to novel individuals with minimal labeled data [65]. To address model uncertainty, Monte Carlo Dropout is applied, providing predictive confidence intervals that enhance reliability and support clinician trust [66] (Figure 1).

### Reasoning Layer: Intelligent Decision-Making for Cognitive Intervention Optimization

The Reasoning Layer enables the transformation of predictive insights into dynamic, personalized intervention strategies through adaptive and explainable decision-making processes [67]. Short-term RL algorithms are used to dynamically optimize immediate intervention plans based on real-time DT feedback, while long-term RL models evolve personalized intervention trajectories to maximize cognitive resilience over extended time horizons [68]. Bayesian probability estimation frameworks adjust risk stratification dynamically by incorporating model uncertainty [69]. Causal inference engines, using methods such as propensity score matching, infer the causal effectiveness of different intervention strategies, supporting evidence-based personalization [70]. Anomaly detection modules continuously monitor physiological and behavioral feature streams to identify early warning signs of cognitive decline, prompting timely interventions [71]. To enhance transparency and clinical interpretability, explainable AI techniques, specifically Shapley additive explanations and local interpretable model-agnostic explanations, are integrated to elucidate both global model behavior and individualized risk predictions [72] (Figure 1).

### Application Layer: Real-Time Cognitive Health Management Through User-Centered Interfaces

The Application Layer enables the delivery of real-time cognitive health insights and intervention guidance to clinicians and patients through intuitive, user-centered interfaces [73]. For health care providers, the system offers interactive dashboards presenting real-time cognitive risk assessments, predictive trajectories, and personalized intervention recommendations [74]. Simulation tools enable clinicians to explore the potential effects of various intervention strategies on cognitive outcomes [75]. For patients, mobile health apps dynamically deliver customized mind-body exercise programs, including Baduanjin, Tai Chi, and Yoga, along with cognitive rehabilitation modules targeting memory, attention, and executive functioning [32-36,76]. Intelligent lifestyle coaching interventions are personalized to optimize sleep hygiene, dietary habits, physical activity, and psychological resilience [77]. Wearable device interfaces facilitate continuous remote monitoring, deliver anomaly alerts, and track adherence to prescribed interventions, closing the feedback loop between prediction, intervention, and outcome [78]. Through these user-centered interfaces, the DT framework enables dynamic, evidence-informed, and individualized cognitive health management across diverse care settings [79] (Figure 1).

### Cloud-Native Infrastructure for Scalable DT Deployment

#### Overview

**Figure 2:**
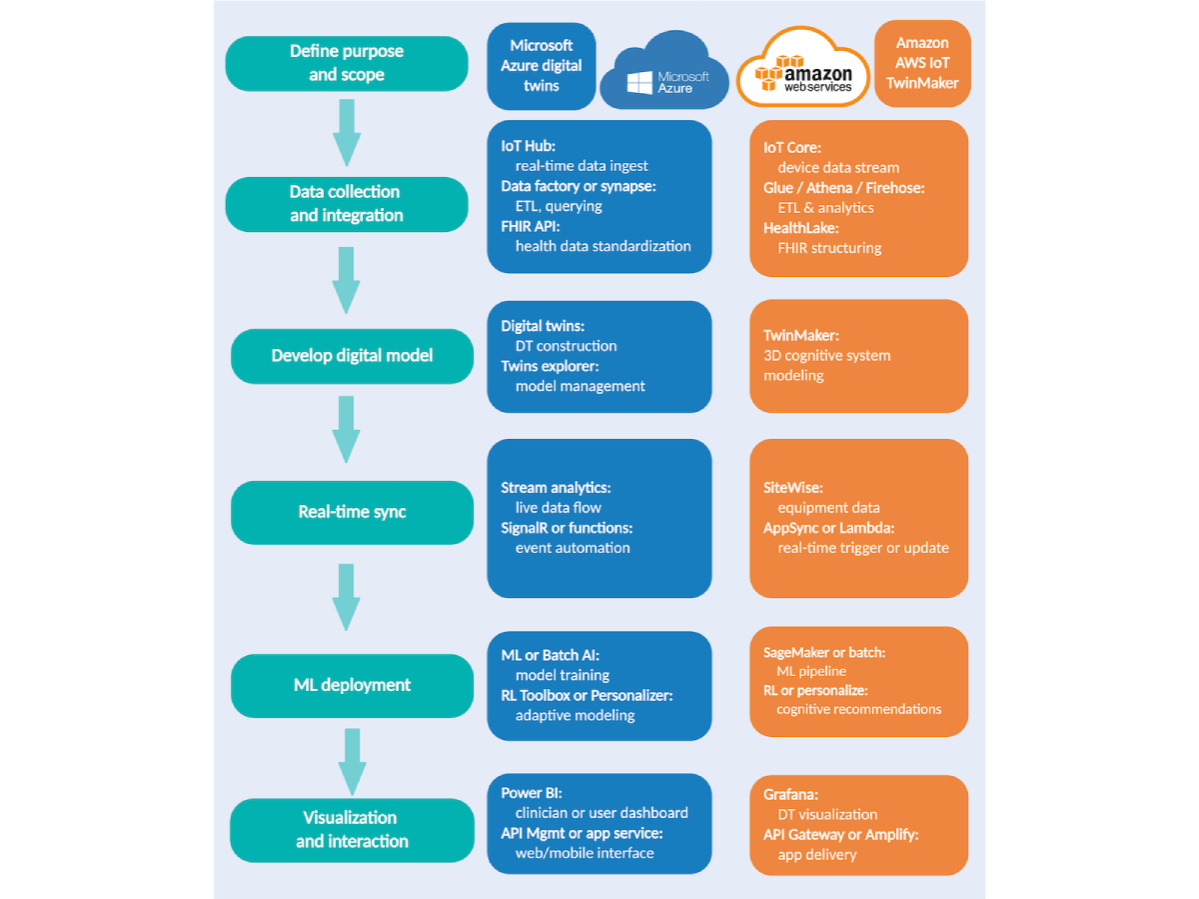
Modular Pipeline for Cloud-Based DT Architecture Using Microsoft Azure and Amazon AWS. Created in BioRender [81], licensed under CC BY 4.0 [17]. AWS: Amazon Web Services; BI: business intelligence; DT: digital twin; IoT: Internet of Things; ML: machine learning.

The Cloud-Native Infrastructure enables scalable, secure, and real-time deployment of cognitive DTs across diverse health care settings by leveraging advanced cloud computing services and architectures [80]. To achieve this, we implemented DT deployments on 2 leading cloud platforms, Amazon Web Services (AWS) and Microsoft Azure, and integrated advanced services to support real-time cognitive simulation and decision-making (Figure 2 [81]). The platform-specific services are provided to make the architecture operationally concrete; the core contribution is the vendor-agnostic design pattern (event-driven orchestration, state management, and closed-loop feedback), which can be implemented on either cloud ecosystem.

Figure 2 [81] outlines the stepwise architecture for digital twin development using cloud-based platforms. Azure and AWS are presented as alternative reference implementations to illustrate platform-to-function mapping, rather than as prescriptive recommendations. There are six modules: defining purpose and scope, data collection and integration, digital model development, real-time data synchronization, machine learning model deployment, and visualization and interaction. Azure-based services are shown in blue, and AWS-based services are shown in orange. IoT-based device integration is supported by Azure IoT Hub and AWS IoT Core. Real-time analytics are enabled by Azure Stream Analytics and Amazon Kinesis. Interactive dashboards for clinicians and patients are built using Power BI and Amazon Managed Grafana.

#### AWS-Based DT Architecture

The AWS-based DT architecture leverages a modular suite of cloud services to support real-time data ingestion, cognitive modeling, and intervention optimization [82]. AWS IoT Core manages secure device connectivity and streaming of physiological and behavioral data [83]. Amazon SageMaker facilitates the training, deployment, and continuous updating of ML models, including CNNs [84], LSTMs [85], and RL agents [86]. AWS Lambda supports event-driven serverless computation, enabling dynamic model updating and real-time anomaly response [87]. Amazon HealthLake structures heterogeneous health data within a FHIR-compatible schema, promoting interoperability across systems [88]. Streaming analytics through Amazon Kinesis enables the continuous monitoring of multimodal data flows and supports immediate recalibration of DT models in response to evolving cognitive states [89] (Figure 2).

#### Azure-Based DT Architecture

The Azure-based DT architecture provides an integrated environment for real-time cognitive health management through Azure DTs, Azure IoT Hub, Azure Synapse Analytics, and Azure ML [90]. Azure DTs facilitates the construction of dynamic cognitive health graphs, mapping real-time changes in physiological, behavioral, and environmental states [91]. Azure IoT Hub ensures secure, scalable ingestion of real-time sensor and mobile app data. Azure Synapse Analytics consolidates large-scale multimodal datasets, supporting complex queries and feature extraction workflows. Azure ML orchestrates the training, serving, and retraining of cognitive state prediction models [66]. Additionally, Azure Stack Edge enables edge computing at the point of care, ensuring data preprocessing and privacy preservation close to the data source [92] (Figure 2).

#### Advanced Cloud Services for Real-Time Cognitive Simulation

Both AWS and Azure infrastructures integrate real-time streaming services to enable continuous cognitive state simulation and immediate intervention adjustment [93]. AWS Kinesis Data Streams and Azure Event Hubs provide scalable, low-latency data streaming capabilities essential for updating cognitive models in real time [94]. These services support anomaly detection, dynamic recalibration of RL agents, and deployment of adaptive intervention recommendations without delay [95]. In both environments, comprehensive security frameworks, encompassing encryption at rest and in transit, identity and access management, and audit trails, are enforced to protect patient data integrity [17,96] (Figure 2).

#### Cloud-Native Compliance Control and Governance

To ensure regulatory alignment and operational transparency, the digital twin architecture can integrate compliance‑management tools from AWS and Azure. AWS Security Hub provides a centralized view of security and compliance posture across services and accounts, supporting automated checks against HIPAA‑related and other industry standards [97]. On Azure, Microsoft Defender for Cloud offers a unified compliance dashboard that evaluates resources against HIPAA‑aligned controls, provides remediation guidance, and supports audit readiness [98]. These governance tools enable continuous compliance monitoring, reduce audit burden, and help ensure that cognitive‑health applications operate within a secure and compliant environment.

### Application Scenarios

#### Early Detection and Real-Time Risk Monitoring in Cognitive Decline

Early recognition of cognitive vulnerability and continuous dynamic monitoring are critical to preventing progression toward overt cognitive impairment [99]. The early detection module proactively identifies individuals at heightened risk by continuously analyzing multimodal physiological and behavioral signals. By detecting deviations from personal baselines before the emergence of clinical symptoms, the system issues timely alerts, enabling clinicians to initiate preventive strategies during the most responsive stages of cognitive decline [100]. The dynamic risk monitoring module ensures real-time surveillance of cognitive status and associated physiological behaviors [101]. Upon detection of significant risk elevation, the system prompts adaptive modifications to cognitive training intensity, exercise prescriptions, or lifestyle interventions, aiming to stabilize cognitive function and prevent crisis events [102]. This integrated approach supports proactive, longitudinal risk management across diverse aging populations (Table 2 and Figure 1).

**Table 2:** Functional modules of the digital twin framework across application domains.

Application domain, functional module^a^			Core objective
**Early detection and continuous risk monitoring**			
	Early detection module	Identify early signs of cognitive decline through anomaly detection and predictive modeling.	
	Dynamic risk monitoring module	Continuously track cognitive and physiological indicators in real time to enable proactive interventions.	
**Personalized intervention and adaptive care optimization**			
	Mind-body exercise recommendation module	Dynamically recommend personalized mind-body interventions (eg, Baduanjin, Tai Chi, and Yoga) based on real-time assessments.	
	Cognitive rehabilitation module	Deliver individualized cognitive training programs targeting memory, attention, and executive function.	
	Lifestyle coaching module	Provide real-time personalized guidance on sleep, nutrition, physical activity, and psychological resilience.	
**Predictive simulation and value-based cognitive care planning**			
	Disease progression simulation module	Simulate cognitive disease trajectories under different intervention scenarios to support personalized care planning.	
	Health economic evaluation module	Evaluate the cost-effectiveness of intervention strategies to support value-based decision-making.	

^a^This table summarizes the seven functional modules of the cognitive digital twin framework, grouped by three major application domains. Modules support early risk detection, real-time intervention adaptation, and predictive planning for value-based cognitive care in aging populations.

#### Personalized Cognitive Intervention and Adaptive Mind-Body Care for Aging Populations

Sustaining cognitive resilience requires interventions that are not only individualized but dynamically adaptable to changing cognitive and physiological states [103]. The Mind-Body Exercise Recommendation Module delivers personalized programs, including Baduanjin, Tai Chi, and Yoga, tailored to the user’s real-time cognitive and physical profile [32,34-36]. Engagement, physiological responses, and health trends continuously inform adjustments to optimize adherence and maximize neuroprotective benefits [104]. The Cognitive Rehabilitation Module offers targeted cognitive training exercises that adapt in difficulty and focus based on ongoing cognitive assessments, aiming to enhance memory, attention, and executive functioning in a progressive, individualized manner [105]. The lifestyle coaching module continuously integrates behavioral data to deliver tailored advice on sleep hygiene, nutritional optimization, physical activity enhancement, and psychological resilience, fostering a holistic approach to cognitive and emotional well-being [106]. Together, these modules form a closed-loop, adaptive system that continuously refines intervention strategies, ensuring dynamic alignment with individual trajectories over time [107] (Table 2 and Figure 1).

#### Predictive Trajectory Simulation and Value-Based Planning for Cognitive Disease Management

Beyond day-to-day management, the DT framework empowers strategic cognitive health planning through predictive modeling and economic evaluation [108]. The Disease Progression Simulation Module enables users and clinicians to visualize projected cognitive trajectories, particularly transitions from MCI to dementia, under alternative intervention strategies, facilitating personalized, evidence-driven decision-making [109]. The Health Economic Evaluation Module assesses the cost-effectiveness of different cognitive health pathways, generating metrics such as incremental cost-effectiveness ratios and quality-adjusted life years [110]. These evaluations support health care providers and policymakers in prioritizing interventions that maximize clinical benefit while ensuring sustainable resource use [111] (Table 2 and Figure 1).

### Ethical Considerations

The continuous cognitive and emotional monitoring enabled by DTs raises profound ethical and privacy challenges [112]. While technical safeguards such as differential privacy, encrypted local processing, and decentralized federated learning are embedded within the framework, true ethical assurance demands more than technical compliance [113]. Transparent, dynamic consent models must be established, empowering individuals to control the collection, use, and sharing of their cognitive data over time [114]. Algorithmic fairness audits and explainability measures must be incorporated to ensure that adaptive decision-making processes are understandable and trustworthy [115]. Furthermore, equitable access to cognitive DT technologies must be proactively designed [116]. Without deliberate attention to cultural sensitivity, digital literacy support, and accessibility accommodations, there is a significant risk that new disparities could emerge, disproportionately affecting marginalized or cognitively vulnerable populations [112]. Ensuring that the benefits of cognitive DTs are widely distributed remains a critical imperative.

## Discussion

### Principal Findings

This study proposes a conceptual AI-driven DT framework that integrates continuous multimodal data acquisition, predictive cognitive modeling, dynamic intervention optimization, and real-time adaptive feedback within a scalable cloud-native architecture. Structured across five synergistic layers, data acquisition, integration, modeling, reasoning, and application, the framework addresses major gaps in early cognitive risk detection, longitudinal surveillance, and individualized intervention in aging populations. Although currently conceptual, this architecture lays a technical and clinical foundation for transitioning cognitive health care from reactive, episodic models to proactive, precision-driven management, positioning cognitive DTs as a transformative modality in future health care ecosystems.

### Aging, Cognitive Health, and the Need for Innovation

The accelerating demographic shift toward aging societies underscores the urgent need for innovation in cognitive health care [117]. MCI and dementia represent leading contributors to disability and dependency among older adults, yet conventional health care models, often reactive and episodic, fail to address the heterogeneous progression and early warning signals of cognitive decline [118]. Fragmented service delivery, limited access to specialized care, and escalating health care costs further exacerbate these challenges [119]. Current interventions rarely incorporate continuous monitoring or real-time personalization, leading to missed opportunities for early intervention and prevention [120]. Emerging technologies such as AI, ML, and DT systems offer transformative opportunities to shift cognitive health care toward dynamic, individualized, and evidence-informed models [121]. By embedding real-time multimodal sensing, predictive analytics, and adaptive decision-making into a continuously evolving DT, our framework aspires to enable proactive cognitive health management at scale [122].

### Transformative Potential of DTs in Cognitive Health and Remote Monitoring

The proposed framework advances the field by introducing several critical innovations. Multimodal data, including EEG, HRV, gait patterns, speech features, sleep architecture, and environmental exposures, are continuously captured through wearable sensors, IoT devices, and mobile apps [123]. These diverse signals are harmonized using FHIR and OMOP standards and integrated through sophisticated cross-modal autoencoders and feature concatenation pipelines, creating unified cognitive state representations [124]. Predictive models incorporate CNNs for spatial feature extraction, LSTM networks for temporal dynamics, and extreme gradient-boosted trees and SVMs for structured clinical data analysis [125]. Dynamic decision-making is achieved via short-term and long-term RL algorithms, enabling immediate adaptation and long-term trajectory optimization [126]. Uncertainty estimation and causal inference modeling further support clinical interpretability and evidence-based intervention planning [127].

Importantly, the system extends the concept of remote patient monitoring beyond traditional vital sign tracking [80]. By capturing dynamic cognitive and behavioral indicators in real-time, the DT continuously assesses cognitive resilience, predicts risk elevations, and recommends personalized adaptive interventions [122]. Cloud-native deployment leveraging federated learning and edge computing ensures that the framework is scalable, privacy-preserving, and responsive across diverse health care settings [128]. Collectively, these advances position cognitive DTs as a next-generation platform for remote monitoring, predictive simulation, and personalized cognitive health optimization [121].

### Technical and Practical Challenges

While the conceptual architecture is robust, substantial technical barriers must be addressed for clinical translation. Seamless multimodal data integration remains challenging, particularly given the asynchronous, noisy, and incomplete nature of real-world data [129]. Variability in device quality, user adherence, and environmental conditions introduces further complexity [80]. Real-time model updating through RL demands computational efficiency, low latency, and scalability across distributed cloud-edge infrastructures, requirements that are not yet fully met by existing health care AI pipelines [130]. Developing lightweight, resource-efficient online learning algorithms capable of dynamic personalization without compromising predictive accuracy will be essential [129]. Additionally, ensuring economic sustainability for continuous cognitive monitoring systems will require careful optimization of data synchronization intervals, computational resource allocation, and infrastructure costs. Real-world deployment in older adults is further constrained by systematic missingness and representativeness gaps and by methodological limits under highly heterogeneous (non-IID) data that motivate personalized federated learning in low-label longitudinal settings.

### Limitations of the Present Framework

Several limitations should be noted. First, this work presents a conceptual and architectural framework, and it has not yet been tested in clinical trials or real-world deployment. As a result, its predictive performance, clinical usefulness, user engagement, and feasibility still need to be established. The models mentioned here, such as CNN, LSTM, and SVM, are included as examples rather than as definitive solutions. In reality, data collected in aging research and care are often messy and complicated, with irregular follow-up, missing values, multimorbidity, and multiple data types. Addressing these challenges will likely require more specialized modeling approaches than those briefly described here.

There are also important technical issues that remain unresolved, including how best to combine asynchronous multimodal data, handle missingness, and optimize RL strategies under real-world conditions. Interpretability will be equally important, and tools such as Shapley additive explanations and local interpretable model-agnostic explanations may need to be applied more thoughtfully across the full modeling pipeline. In addition, the framework still needs to be tested across populations with different cognitive baselines, medical comorbidities, socioeconomic circumstances, and cultural backgrounds. Finally, although the current framework focuses on mind-body practices, cognitive rehabilitation, and lifestyle support, a more complete digital twin for cognitive care will likely need to incorporate broader treatment domains, including pharmacological and psychosocial interventions. Together, these limitations do not diminish the value of the framework, but instead point to the key areas that future work should address.

### Future Directions

Looking ahead, future work should focus on building and testing cognitive digital twin systems step by step. This includes usability testing in diverse older adult populations, as well as longitudinal studies to determine whether these models can meaningfully predict outcomes and support adaptive interventions in real-world settings. Further technical development will also be needed to handle noisy, incomplete, and multimodal data while maintaining interpretability and efficiency.

Just as importantly, future research should address ethical and governance issues, including consent, privacy, fairness, and participant involvement in data use. Over time, it may also be valuable to expand cognitive digital twins beyond cognitive decline alone to include emotional well-being, frailty, multimorbidity, and social factors that shape aging and health.

### Conclusions

In this study, we present a conceptual AI-driven, multimodal digital twin framework for precision cognitive care in aging populations. By supporting continuous monitoring, early risk detection, personalized intervention, and adaptive learning, the framework offers a proactive and patient-centered approach to cognitive health. Although empirical validation and further technical development are still needed, this work provides a useful foundation for future efforts to make cognitive care more personalized, responsive, and sustainable.

## Abbreviations

AI: artificial intelligence
AWS: Amazon Web Services
CNN: convolutional neural network
DT: digital twin
EEG: electroencephalography
FHIR: Fast Healthcare Interoperability Resources
HIPAA: Health Insurance Portability and Accountability Act
HRV: heart rate variability
MCI: mild cognitive impairment
ML: machine learning
OMOP: Observational Medical Outcomes Partnership
LSTM: long short-term memory
RL: reinforcement learning
SVM: support vector machine

## Data Availability

This study is conceptual in nature and does not report or analyze any datasets. As no new data were generated, data sharing is not applicable.
